# Costimulatory Molecules OX40 and OX40L Upregulation in Oral Squamous Cell Carcinoma: A Blood-Based Study

**DOI:** 10.1055/s-0042-1760375

**Published:** 2023-01-18

**Authors:** Aliya Irshad Sani, Zila Rubab, Shumaila Usman, Syed Zaryab Ahmed, Sadia Arif

**Affiliations:** 1 Department of Biochemistry, Ziauddin University, Karachi, Pakistan; 2 Department of Molecular Medicine, Ziauddin University, Karachi, Pakistan; 3 Department of Pathology, Ziauddin University, Karachi, Pakistan

**Keywords:** OX40, OX40L, immunotherapy, oral squamous cell carcinoma, costimulatory molecules

## Abstract

**Objectives** This research aimed to determine OX40 and OX40L mRNA expression in blood samples of naive oral squamous cell carcinoma (OSCC) patients in different histological grades and clinical stages. The in silico analysis was performed using the STRING database for functional association and a better understanding of the interactions of OX40 and its ligand with other proteins.

**Materials and Methods** In this study, we recruited 141 newly diagnosed patients of OSCC. Levels of OX40 and OX40L mRNA expression were explored using real-time quantitative polymerase chain reaction. An in silico tool was also utilized to evaluate the OX40/OX40L interactome.

**Results** The results showed higher OX40 expressional levels in the late stage (23-fold) compared with the early stage (8.5-fold) ( *p* = < 0.001). A similar trend was seen in OX40L mRNA expression, revealing a fold change of 5.8 in the early stage in comparison to 9.9-fold change in the late stage ( *p* = < 0.001). Overexpression of OX40 and OX40L was found in different histological grades ( *p* = 0.005 and *p* = < 0.001, respectively). Overexpression of OX40 and OX40L was detected in habits such as smoking and paan intake, whereas statistically significant upregulation was observed in the cheek, lip, and alveolus tumors. However, there was no substantial difference in OX40 and OX40L expression based on age or gender. The functional interactions, that is, interactomes of OX40 and OX40L with other proteins have been determined by in silico analysis.

**Conclusion** Based on current study findings, despite OX40 and OX40L upregulation in newly diagnosed OSCC patients, it is speculated that the physiological function of these molecules is altered due to immune system exhaustion.

## Introduction

In 2020, the global incidence of oral squamous cell carcinoma (OSCC) was 377,713 with 177,757 fatalities reported worldwide. 1 It is also the most frequent malignancy among Pakistani men, with a twofold higher incidence in men than in women. OSCC originates from the malignant transformation squamous epithelial lining of the oral cavity, including the lip, tongue, and cheeks. 2 The main culprits in the occurrence of OSCC are the consumption of chewable tobacco products, alcohol, cigarette smoking, sheesha smoking, and human papillomavirus infections. 3 OSCC can manifest as a hard fixed fungating mass with local invasion, a nonhealing ulcer with uneven, indurated, rolled borders, or an exophytic broad-based mass. 4 5

Oral cancer is managed surgically, chemotherapy, and radiation. 6 The 5-year survival remained ∼50% in OSCC patients, despite the availability of multipronged treatment strategies. Moreover, invasive surgical treatments may also lead to a lower quality of life. There are no recognized guidelines or diagnostic instruments for early identification of OSCC to date. The standard screening includes a routine oral cancer examination in at-risk individuals. However, these methods have been unable to make an early and accurate diagnosis, and detection is difficult until cancer has progressed. 7 8

Presently, newer avenues in oral cancer treatment are being explored, such as CyberKnife radiosurgery and enhanced drug delivery via nanoparticles. 8 9 10 Research is also being conducted into devising diagnostic and therapeutic molecules against cancers in an effort to improve outcomes, with an emphasis on “Biological Hallmarks of Cancer,” one of which is immune system surveillance evasion. 11 Newer immunotherapeutic compounds are being synthesized and tested to augment antitumor activity either as monotherapy or in conjunction with existing therapies in a multitude of malignancies. Although the experimental trials have yielded encouraging outcomes, the challenge of autoimmunity and resistance endures. 12

In the past two decades, scientists are focusing on enhancing antitumor activity by devising therapeutic agents stimulating immune costimulatory molecules. The OX40, that is, “ *tumor necrosis factor receptor superfamily, member 4* ” (CD134, TNFRSF4) and its ligand OX40L,” “ *tumor necrosis factor (ligand) superfamily, member 4* ” (OX40L, CD252, TNFSF4), are fundamental in augmenting the immune response against cancer cells. The expression of OX40 is reported primarily on T cells, whereas OX40L expression is predominantly on antigen presenting cells. Studies on OX40 and its ligand gene and protein expression in various cancers have revealed varying biological behavior and prognostic association, 13 while there is a paucity of data on OX40 and OX40L mRNA expression levels in newly diagnosed OSCC patients. Therefore, the current study pursues to investigate OX40 and its ligand gene expression in blood samples of newly diagnosed OSCC patients in connection to different clinical and pathological characteristics. In addition, we used a bioinformatic tool called the STRING database to elucidate OX40/OX40L interactions with other closely involved proteins to get insight of biological processes and molecular functions that might have role in the immunity against cancer or in favor of cancer progression.

## Materials and Methods

This study was cross-section and the sampling was performed from September 2020 to May 2021 via consecutive sampling technique. The study was granted approval by the ethics committee of Ziauddin University (ERC # 2410720ASBC). In this study, 141 newly diagnosed biopsy-proven OSCC patients with no prior cancer-related treatment were recruited. All subjects included in this study were included from either the Dental OPD of Ziauddin Hospital or the Maxillofacial Surgery OPD of Abbasi Shaheed Hospital. Once enrolled and after the informed consent, the pro forma was filled out. Clinical staging and histological grade were determined using investigations such as computed tomography scan imaging and biopsy reports. To separate the buffy coat, 5 mL of blood were taken and decanted into EDTA tubes, then centrifuged at 2,000 rpm for 10 minutes. The buffy coat was then transferred to Eppendorf tubes and stored at −80°C for RNA extraction using TRIzol (Thermo Fischer Scientific) extraction method. The RNA yield was ascertained with Multiskan spectrophotometer. The total RNA isolated was reverse transcribed to cDNA by using RevertAid First Strand cDNA Synthesis Kit (Thermo Fischer Scientific), according to the manufacturer's instruction.

The 10 μL of quantitative polymerase chain reaction (PCR) was prepared by taking 0.5 μg cDNA, forward and reverse primer 2.5 μL each, adding 1 μL master mix and nuclease-free water. The thermal cycle was programmed as follows: initial denaturation for 4 minutes at 95°C, denaturation for 30 seconds at 95°C, annealing for 30 seconds at 57°C, and extension for 10 seconds at 72°C with total of 40 cycles. Quantification of OX40 and OX40L was evaluated by following formulas after normalizing the cycle threshold (CT) with *GAPDH* (housekeeping gene):

ΔCT (delta CT) = CT _gene of interest_ − CT _GAPDH_

ΔΔCT (delta delta CT) = ΔCT _diseased_ − ΔCT _control_

Fold change = 2^ΔΔCT.

The primers used in this study were self-designed by using “Primer designing tool,” ( https://www.ncbi.nlm.nih.gov/tools/primer-blast/ ), given in Table 1 .

**Table 1:** List of primers

S. no.	Gene	Accession number	Primer sequence	Annealing temperature	Product size (bp)
1	GAPDH (glyceraldehyde-3 phosphate dehydrogenase)	BC-025925	(Forward) CCAGAACATCATCCCTGCCT	58°C	185
			(Reverse) CCTGCTTCACCACCTTCTTG		
2	OX40 (tumor necrosis factor receptor superfamily, member 4)	XM-011509964	(Forward) CAAGCGTGGACTTGACTGTG	58°C	155
			(Reverse) GGTCCCTGTCCTCAGATT		
3	OX40L (tumor necrosis factor superfamily, member 4)	BC-041663	(Forward) GTCTGGGATGTGATGCTTT	58°C	208
			(Reverse) GTGTTGCTTTGCCTGTCTGT		

The interactome analysis of OX40/OX40L was performed by the software STRING version 11.5 database tool ( http://stringdb.org/). ^14^ The OX40 was used as an input, and the settings were used to generate the first shell of interactions, which had only nine interactions along with OX40 and a confidence interval of 0.9. The generated interactome determines functionally and physically closely associated proteins with OX40 and may give insight into the complexity of biological processes.

### Statistical Analysis

The collected data were analyzed by using SPSS version 25. The categorical data were presented as frequency and percentage. OX40 and OX40L gene expression in different stages and histological grades were analyzed by using Kruskal–Wallis' test followed by pairwise comparisons. Gene expression of OX40 and OX40L were correlated by using Spearman's correlation.

## Results

The average age of the healthy individuals, that is, controls ( *n* = 10) was 42 ± 5.8 years, while the mean age of the OSCC patients included in the study was 50.62 ± 12.7 years. Table 2 lists the demographic and clinicopathological characteristics of OSCC patients.

**Table 2:** Demographic of OSCC patients

Characteristics	Frequency	Percentage (%)
Ethnicity		
Urdu speaking	68	48.2
Sindhi	17	12.1
Punjabi	21	14.9
Pathan	8	5.7
Balochi	10	7.1
Others	17	12.1
Occupation		
Business	6	4.3
Housewife	11	7.8
Driver	11	7.8
Shopkeeper	12	8.5
Retired	12	8.5
Mechanic	13	9.2
Labor	23	16.3
Others	53	37.5
Habits		
Smoking	50	33.1
Alcohol	6	4
Paan	31	20.5
> 1 habit	38	25.2
Naswar	3	2
None	13	8.6

Abbreviation: OSCC, oral squamous cell carcinoma.

**Fig. 1:**
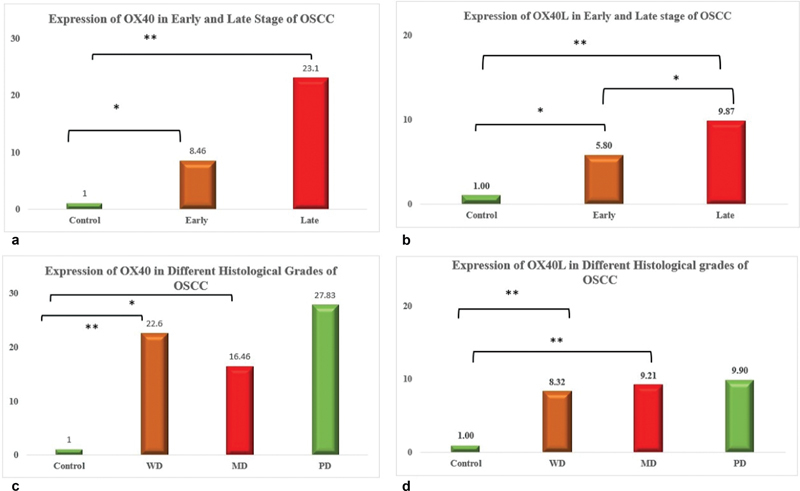
**Expression of OX40 and OX40L in OSCC.** ( **a** ) Expression of OX40 in early- and late-stage OSCCs, *p* -value of <0.001. Pairwise comparison revealed control and early-stage *p* -value = 0.018; control and late-stage *p* -value = < 0.001. ( **b** ) Expression of OX40L in early- and late-stage OSCCs, *p* -value of <0.001. Pairwise comparison revealed control and early-stage *p* -value = 0.024; control and late-stage *p* -value = < 0.001; early-stage and late-stage *p* -value = 0.01. ( **c** ) Expression of OX40 in different histological grades of OSCC, *p* -value = 0.005. Pairwise comparison revealed control and WD *p* -value = 0.002; control and MD *p* -value = 0.006. ( **d** ) Expression of OX40L in different histological grades of OSCC, *p* -value of <0.001. Pairwise comparison revealed control and WD *p* -value = < 0.001; control and MD *p* -value = < 0.001. (Statistical test applied Kruskal–Wallis.) MD, moderately differentiated; OSCC, oral squamous cell carcinoma; PD, poorly differentiated; WD, well differentiated.

Real-time quantitative PCR was performed to determine the expression of costimulatory immune modulators, OX40, and OX40L mRNA in blood samples of naïve oral cancer patients. The relative quantification of OX40 mRNA levels according to clinical staging revealed upregulation in early stage (8.5-fold) and late stage (23-fold) compared with controls, which was statistically significant ( *p* = 0.001). On the other hand, relative quantification of OX40L mRNA levels revealed upregulation in early stage (9-fold) and late stage (18-fold) and was found to be statistically significant *p* = < 0.001. Expressional levels were also analyzed based on the histological grading. The relative quantification of OX40 mRNA levels showed higher expression values in well-differentiated tumors (22.6-fold) compared with moderately differentiated (16.46-fold) and poorly differentiated (27.8-fold) was found to be statistically significant, *p* = 0.005. On the contrary, relative quantification of OX40L mRNA levels revealed upregulation in well-differentiated tumors (8.3-fold) compared with moderately differentiated (9.2-fold) and poorly differentiated (9.9-fold) was found to be statistically significant, *p* = < 0.001 as illustrated in Fig. 1 . The expression of OX40 and OX40L did not significantly differ between patients who were older than 50 years and those who were younger than 50 years, nor did it differ significantly between the genders ( Table 3 ).

**Table 3:** Relative OX40 and OX40L gene expression in OSCC patients

Variables	OX40 mRNA expression	*p* -Value	OX40L mRNA expression	*p* -Value
Age				
< 50 y	19.74 (47.52)	0.754	8.91(12.22)	0.821
> 50 y	17.165 (75.87)		9.39 (15.29)	
Gender				
Male	20.39 (66.03)	0.46	8.32(13.79)	0.993
Female	4.67 (50.45)		9.89(13.04)	

Abbreviation **: OSCC, oral squamous cell carcinoma.**

**Fig. 2:**
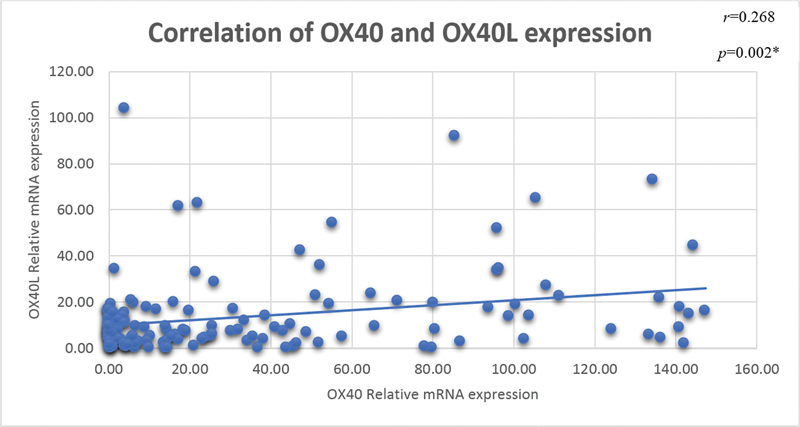
Correlation of relative gene expression of OX40 and OX40L in oral squamous cell carcinoma patients.

**Fig. 3:**
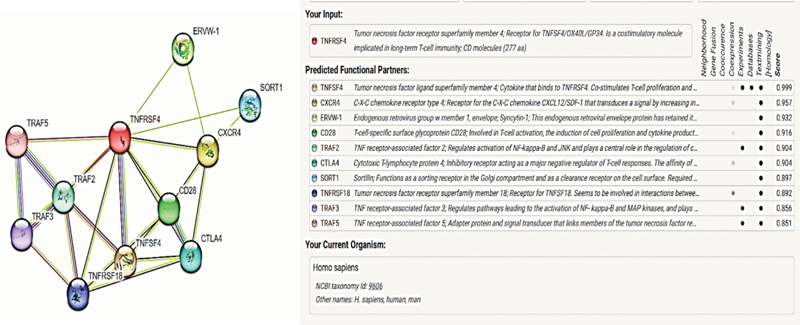
Protein–protein interaction network of OX40 and OX40L using STRING software.

The relative expression of OX40 and OX40L according to the site and habits is presented in Table 4 . Overexpression of OX40 and OX40L was also observed in OSCC patients with more than one habit. The expression of OX40 and OX40L in different sites of tumors was detected to be upregulated with significant differences in tumors of cheek, lip, and alveolus. In OSCC patients, there was a weak positive correlation between OX40 mRNA expression and OX40L mRNA expression ( *r* = 0.268, *p* = 0.002) depicted in Fig. 2 . Using bioinformatics tool, the STRING database program, the protein–protein interaction (PPI) of OX40 in humans was determined. OX40's interactome revealed nine well-known interactors in addition to its ligand. The strong interaction was observed for CXCR-4, ERVW-1, CD28, TRAF-2, CTLA-4 other than OX40L (TNFSF4) at confidence score of ≥ 0.9 as depicted in Fig. 3 with PPI enrichment *p* -value of <0.005.

**Table 4:** Relative OX40 and OX40L gene expression in OSCC patients according to different habits and tumor sites

	*n*	OX40 fold change	*p* -Value	OX40L fold change	*p* -Value
Habits					
Smoking	50	21.73 (62.64)	0.015	7.5(13.86)	<0.001
Alcohol	6	14.85(35.13)		4.96 (4.78)	
Paan	31	13.59 (46.29)		15.09 (14.67)	
Naswar	3	16.05		7.4	
> 1 habit	38	16.35 (100.47)		81.2 (11.40)	
None	13	30.06 (133.90)		9.89 (16.27)	
Sites of tumor					
Cheek	96	21.27 (67.55)	0.014	8.97 (13.73)	<0.001
Tongue	12	14.69 (43.96)		4.56 (12.37)	
Alveolus	9	16.05 (97.64)		14.17 (15.84)	
Lip	18	16.96 (96.62)		11.80 (14.29)	
palate	6	18.21 56.41)		5.76 (9.92)	

Abbreviation: OSCC, oral squamous cell carcinoma.

## Discussion

The current study demonstrates that OX40 and OX40L were upregulated in both the early and late stages of OSCC. The gene expression levels in OSCC increased from early to later stages. This surge in costimulatory molecules in later stages is most likely a marker of tumor-associated inflammation having a role in cancer cell proliferation in conjunction with the patient's exhausted immune system, implying a poor prognosis. The increasing upregulation of OX40 has been also reported in breast carcinoma with advancing disease and poorer outcomes. 15 Similarly, higher OX40 and OX40L expression has been linked to later stages and worse outcomes in head and neck cancer. 16 17

The state of inflammation, which might be heterogeneous at different tumor locations, can influence OX40 and OX40L expression levels. Our findings suggest that while OX40 and OX40L expression levels are upregulated, other factors, such as the host environment and immunosuppressive mediators such as elevated PD1, CTLA-4, and significantly reduced IL-2, are deterring costimulatory molecules from functioning normally, leading to disease progression. 18 19 20 Another aspect is the expression of OX40 on Tregs is also important because of its controversial role in the tumor immunity. Abundance of Tregs was observed in later stages of OSCC, and this could explain overexpression of costimulatory molecules in the blood. 20 Moreover, infiltration of tumors by Tregs promotes growth and metastasis by inhibiting antitumor immunity, the involvement of Tregs in tumor progression has been studied extensively, and similar results were found in many other tumor types. It is reasonable to hypothesize that the higher levels of Tregs in OSCC similarly contribute to tumoral immune escape and indicate reduced survival. 21 22 The conflicting results indicate that OX40 signaling may regulate Tregs in multiple ways, meaning that the effect of OX40 agonist on Tregs may differ depending on various factors, such as cytokines and other stimulation.

The present study results revealed statistically significant difference of OX40 and OX40L in different histological grades of OSCC. However, most of the studies have reported their expression in tumor-infiltrating lymphocytes present in the tumor microenvironment. Scarce data are available on their expression in different histological grades. Researches in breast, hepatic, and colorectal carcinomas have exhibited no significant association of OX40 and OX40L positivity with the histological grades. 15 23 24 Another study in colorectal carcinoma demonstrated higher expression in 87.1% cases of moderately differentiated tumors; however, no significant association was observed. 25 The serum levels of OX40 and its ligand were not associated with histological grading in OSCC. 26 27

The tumor sites of cheek, alveolus, and lip showed significant overexpression of these costimulatory molecules as well as in patients who had habits such as paan consumption and smoking. Evidence is there that nicotine and areca nut consumption can lead to chronic inflammation by increasing macrophages and lymphocytes at the site of the tumor as well as elevated C-reactive protein levels. 28 29 30 The role of OX40/OX40L is reported in development of chronic inflammation due to nicotine exposure. 31 There is no such study which has evaluated the expression of OX40/OX40L in relation to different habits and tumor site in OSCC.

The interactome shows the PPIs of OX40 and OX40L with other mediators and shows proteins that need to be explored for better understanding of T cell biology and the therapeutic agents based on OX40 and OX40L against malignancies. The interactome reveals the core protein, CXCR4, by exhibiting second highest confidence score of 0.957 after OX40L confirms its maximum interaction with OX40. Other than CXCR4, ERVW1, CD28, TRAF2, and CTLA4 were mediators involved in T cell activation as well as immunoglobulins regulation have been found to strongly interact with OX40. Functional analysis of interactome showed biological processes such as negative and positive regulation and differentiation of regulatory T cells, regulation of immunoglobulin-mediated immune reactions and their secretions. For molecular functional aspect, cytokine receptor binding and their activity as well as growth factor receptor binding and their activity were the known molecular mechanisms in which the interactome proteins of OX40 were engaged. 14 The purpose was to establish that immunological processes are complicated and function through intricate networks, and that any component of the pathway that is dysregulated might influence others.

The therapeutic agents based on OX40 and OX40L are in clinical trials. Expressional levels of OX40 and OX40L may predict the effectiveness of such agents. However, we suggest that the role of other immune modulators linked to the OX40 and OX40L should also be evaluated as we are of the opinion that the upstream modulators need to be activated first to enhance the function of OX40-based agents. Such approach may aid in devising a right combinational immunotherapy and clinical success. Such approach can be easily acquired by predicted pathways as demonstrated in the interactome and using bioinformatic tools.

The main strength of the study was that the OX40 and OX40L levels were measured in the blood representing the whole-body milieu. However, source of OX40 and OX40L could not be determined. We recommend, OX40 and OX40L gene expressional levels should be estimated in larger cohort with pretreatment and posttreatment cancer patients for comparative analysis along with the phenotyping of cells expressing these costimulatory molecules because of their controversial role in anticancer immunity.

## Conclusion

This study provides the basis for future research for evaluating the blood OX40 and OX40L expressional levels, as an alternative to tumor biopsies. The majority of oral cancer patients suffer with devastating disfigurement, speech and swallowing impairment after surgery and radiotherapy. Immunotherapy is an emerging treatment modality but still needs exploration of mediators that can be targeted to achieve radical cure. Higher expressional levels of OX40 and OX40L in the blood can represent the whole-body milieu and can be utilized as an alternative to repetitive tumor biopsies to evaluate the efficacy of OX40-based trials. However, if higher expression represents immune exhaustion, can these OX40-based agonists be beneficial?
